# Decreased expression of IDH1-R132H correlates with poor survival in gastrointestinal cancer

**DOI:** 10.18632/oncotarget.12039

**Published:** 2016-09-15

**Authors:** Jieying Li, Jianfei Huang, Fang Huang, Qing Jin, Huijun Zhu, Xudong Wang, Meng Chen

**Affiliations:** ^1^ Department of Pathology, Affiliated Hospital of Nantong University, Nantong, Jiangsu, China; ^2^ Department of Laboratory Medicine & Department of Clinical Tissue Bank, Affiliated Hospital of Nantong University, Nantong, Jiangsu, China; ^3^ Department of Molecular, Cellular and Biomedical Sciences, University of New Hampshire, Durham, NH, USA

**Keywords:** gastrointestinal cancer, IDH1-R132H, prognosis, tissue microarray, immunohistochemistry

## Abstract

Isocitrate dehydrogenase (IDH1) is an NADP-dependent enzyme that catalyzes the decarboxylation of isocitrate to alpha-ketoglutarate. The IDH1-R132H mutation predicts a better clinical outcome for glioma patients, and the expression of IDH1-R132H correlates with a favorable outcome in patients with brain tumors. Here, we investigated IDH1-R132H expression in both gastric (n=526) and colorectal (n=399) tissues by performing immunohistochemistry analyses on tissue microarrays. We also tested whether IDH1-R132H expression correlated with various clinical parameters. In both gastric and colorectal cancer, expression of IDH1-R132H was associated with tumor stage. Patients with low IDH1-R132H expression had a poor overall survival. Our data indicate that IDH1-R132H expression could be used as a predictive marker of prognosis for patients with gastrointestinal cancer.

## INTRODUCTION

Gastric cancer is the third leading cause of death from malignancy worldwide [1]. In developing countries, GC accounts for over 70% of all cancers, with more than half of GC cases occurring in eastern Asia [2]. *Helicobacter pylori* infection is a strong risk factor for GC [3, 4]. Although the mortality rate of GC patients has decreased due to improvements in surgical care [5], in Asia, GC still constitutes a heavy economic burden [6]. For example, in China GC is the third leading cause of cancer-related deaths, affecting 32 per every 100,000 males and 13 per every 100,000 females [7].

Colorectal cancer (CRC) is also the third most common tumor among men and the second among women [8]. Although the morbidity of CRC is generally lower in Asia than in the Western world [9, 10], the incidence of colon cancer continues to increase in China [11]. CRC is the fifth most common cause of cancer-related deaths, affecting 14 per every 100,000 people in China [12, 13].

Gastrointestinal cancer results from various factors, including environmental factors and specific genetic alterations that lead to a loss of tumor suppressor genes or to deregulated activity of oncogenes [14–16]. Studying novel molecular prognostic markers might help to elucidate the molecular mechanisms underlying gastric carcinoma. As a member of the IDH enzymes family, cytosolic NADP-dependent isocitrate dehydrogenase (IDH1) is located on 2q33.3 and localizes to peroxisomes and the cytoplasm [17–20]. IDH1 provides needed cytosolic NADPH and regulates its activity through the cholesterol and fatty acid biosynthesis pathways [21]. IDH1 mutations affect cellular metabolism and are often present in gliomas, chondrosarcomas, and acute myeloid leukemias. The most frequent mutation of IDH1 is the R132H mutation, which leads to the replacement of arginine by histidine at codon 132 in the enzymatic active site [22]. This mutation correlates with a positive clinical outcome for patients with glioma [23] and brain tumors [24, 25]. In this study, we used immunohistochemistry (IHC) analysis and tissue microarrays (TMA) to investigate IDH1-R132H expression in malignant gastrointestinal cancer and adjacent normal tissues. We also examined the relationship between IDH1-R132H expression and clinical parameters and overall survival (OS) in gastrointestinal cancer patients.

## RESULTS

### Expression of IDH1-R132H in gastrointestinal tissues

**Figure 1:**
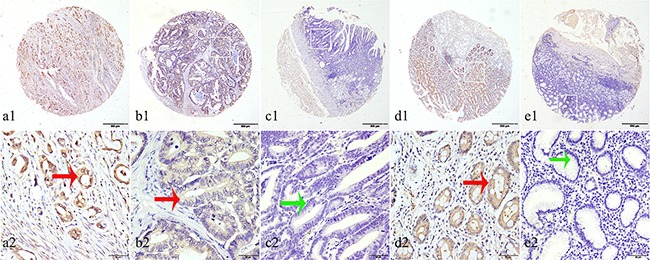
Representation patterns of IDH1-R132H protein expression in gastric benign and malignant tissues in TMA sections **A.** Top row, representative images of immunohistochemical analysis of IDH1-R132H protein in GC tissues. Bottom row, magnified images of the insert boxes in the top row. Red arrows indicate positive IDH1-R132H protein expression and green arrows indicate negative IDH1-R132H protein expression. GC with high IDH1-R132H protein expression. **B.** GC with low IDH1-R132H protein expression. **C.** Intraepithelial neoplasia of gastric mucosa with no IDH1-R132H expression. **D.** Intestinal metaplasia with high IDH1-R132H protein expression. **E.** Chronic gastritis with no IDH1-R132H protein expression. Top row, original magnification ×40 (bar = 500 μm); bottom row, original magnification × 400 (bar = 50 μm).

**Figure 2:**
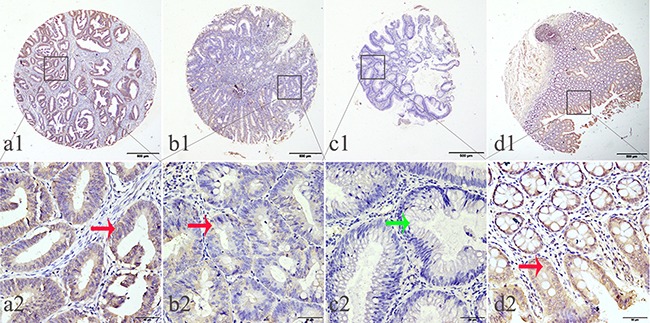
Representation patterns of IDH1-R132H protein expression in colorectal benign and malignant tissues in TMA sections **A.** Top row, representative images of immunohistochemical analysis of IDH1-R132H protein in CRC benign and malignant tissues. Bottom row, magnified images of the insert boxes in the top row. Red arrows indicate positive IDH1-R132H protein expression and green arrows indicate negative IDH1-R132H protein expression. CRC with high IDH1-R132H protein expression. **B.** CRC with low IDH1-R132H protein expression. **C.** Low-grade intraepithelial neoplasia with no IDH1-R132H expression. **D.** Normal surgical margin of CRC with high IDH1-R132H protein expression. Top row, original magnification ×40 (bar = 500 μm); bottom row, original magnification × 400 (bar = 50 μm).

We analyzed gastric and colorectal tumors for IDH1-R132H expression. IDH1-R132H expression was examined at various levels, mainly in the cytoplasm of gastrointestinal cells (Figure 1–2). The cutoff point was defined in accordance with OS in gastric and colorectal tumors by the X-tile software. Here, 120 was selected as the cutoff point for IDH1-R132H in both gastric and colorectal tumors; scores from 0 to 120 were deemed as low expression while scores from 121 to 300 were considered as high expression.

Low IDH1-R132H expression was found in 62.38% (257/412) of GC samples and 39.06% (75/192) of CRC samples. In both cases, the expression of IDH1-R132H was lower than in normal surgical margin tissues and benign tissues (X^2^=17.3833, *P*=0.004; X^2^=18.9286, *P*=0.001; respectively) (Table 1).

**Table 1:** IDH1-R132H expression in gastrointestinal benign and malignant tissues

Characteristic	n	IDH1-R132^−^H−	IDH1-R132^+^H+	Pearson χ^2^	*P*
Stomach				17.3833	0.004*
Chronic gastritis	13	6(46.15)	7(53.85)		
Intestinal metaplasia	12	6(50.00)	6(50.00)		
Low-grade intraepithelial neoplasia	13	7(53.85)	6(46.15)		
High-grade intraepithelial neoplasia	34	18(52.94)	16(47.06)		
Cancer	412	257(62.38)	155(37.62)		
Surgical margin	42	13(30.95)	29(69.05)		
Colon and Rectum				18.9286	0.001*
Chronic colitis	17	6(35.29)	11(64.71)		
Low-grade intraepithelial neoplasia	43	18(41.86)	25(58.14)		
High-grade intraepithelial neoplasia	19	13(68.42)	6(31.58)		
Cancer	192	75(39.06)	117(60.94)		
Surgical margin	128	30(23.44)	98(76.56)		

IDH1-R132H^+^ represents high IDH1-R132H expression; IDH1-R132H^−^ represents low IDH1-R132H expression.

* P<0.05.

**Figure 3:**
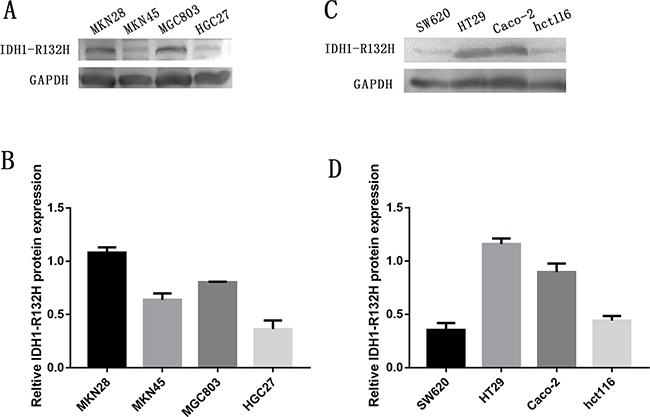
Expression of IDH1-R132H in four GC cell lines and four CRC cell lines **A.** Western blot showing protein levels of IDH1-R132H in GC cell lines with GAPDH as a loading control. **B.** Intensity of IDH1-R132H quantified by densitometry (software: Image J, NIH). Data are reported as mean±SD (n =3). **C.** Western blot showing protein levels of IDH1-R132H in CRC cell lines with GAPDH as a loading control. **D.** Intensity of IDH1-R132H quantified by densitometry (software: Image J, NIH). Data are reported as mean±SD (n =3).

To further verify our findings, we used western blot analysis to measure IDH1-R132H protein expression in four human GC cell lines and four human CRC cell lines. IDH1-R132H was highly expressed in MGC803 cells, MKN28 cells, HT-29, Caco-2, while MKN45 cells, HGC27 cells, hct-116, SW620 had no obvious expression of p42.3 (Figure 3).

### Association of IDH1-R132H expression with clinical parameters in gastrointestinal cancers

We found that in GC, low expression of IDH1-R132H was correlated with tumor stage (X^2^=13.1516, *P*=0.041) and lymph node metastasis (X^2^=12.4282, *P*=0.006). However, we observed no significant correlation between IDH1-R132H expression and other clinical parameters, including sex, age, histology, differentiation, preoperative CEA level, and preoperative CA19-9 level (Table 2). In CRC, low IDH1-R132H expression was correlated with location (X^2^=4.3688, *P*=0.037), and which in colon is higher than it in rectum differentiation (X2=4.5562, P=0.033). Besides, IDH1-R132H expression was also related with tumor stage (X^2^=13.1516, *P*=0.004), lymph node metastasis (X^2^=9.6676, *P*=0.022) and distant metastasis (X^2^=7.4030, *P*=0.007). On the other hand, we detected no correlation between IDH1-R132H expression and gender, age, histology, preoperative CEA level, and preoperative CA19-9 level (Table 3).

**Table 2:** Association of high IDH1-R132H expression with clinicopathological characteristics in gastric cancer patients

Characteristic	n	IDH1-R132H^−^	IDH1-R132H^+^	Pearson χ^2^	*p*
Total	412				
Gender				0.1381	0.710
Male	302	190(62.91)	112(37.09)		
Female	110	67(60.91)	43(39.09)		
Age				0.2391	0.625
<60	147	94(63.95)	53(36.05)		
≥60	265	163(61.51)	102(38.49)		
Histological type				8.8793	0.064
Tubular	354	217(61.30)	137(38.70)		
Mixed (Tubular and mucinous)	12	7(58.33)	5(41.67)		
Mucinous	21	14(66.67)	7(33.33)		
Signet ring cell	15	14(93.33)	1(6.67)		
Others^a^	10	5(50.00)	5(50.00)		
Differentiation				3.4498	0.178
Well	11	4(40.00)	7(60.00)		
Middle	115	68(58.97)	47(41.03)		
Poor	227	144(65.04)	83(34.96)		
Others^b^	58	40	18		
TNM stage				13.1516	0.041*
0+Ia	24	12(50.00)	12(50.00)		
Ib	49	25(51.02)	24(48.98)		
IIa	96	52(54.17)	44(45.83)		
IIb	59	37(62.71)	22(37.29)		
IIIa	75	53(70.67)	22(29.33)		
IIIb	69	49(71.01)	20(28.99)		
IIIc+IV	40	29(72.50)	11(27.50)		
T				2.1390	0.544
Tis+ T1	35	18(51.43)	17(48.57)		
T2	91	56(61.54)	35(38.46)		
T3	252	161(63.89)	91(36.11)		
T4	34	22(64.71)	12(35.29)		
N				12.4282	0.006*
N0	176	79(51.63)	74(48.37)		
N1	81	53(66.25)	27(33.75)		
N2	93	56(68.29)	26(31.71)		
N3	92	69(71.13)	28(28.87)		
M				1.3387	0.247
M0	383	236(61.62)	147(38.38)		
M1	29	21(72.41)	8(27.59)		
Preoperative CEA, ng/m1				1.3118	0.252
≦5	156	104(66.67)	52(33.33)		
> 5	60	35(58.33)	25(41.67)		
Unknown	196	118	78		
Preoperative CA199, U/ml				0.5120	0.474
≦37	156	113(64.20)	63(35.80)		
> 37	60	24(70.59)	10(29.41)		
Unknown	202	120	82		

* *P*<0.05;

a others: papillary adenocarcinoma, 3 cases; adeno-squamous carcinoma, 3 cases; squamous cell carcinoma, 2 cases; neuroendocrine carcinoma,1 case; carcinoid,1 case;

b others: besides tubular.

**Table 3:** Association of high IDH1-R132H with clinicopathological characteristics in colorectal cancer patients

Characteristic	n	IDH1-R132H^−^	IDH1-R132H^+^	Pearson χ^2^	*P*
Total	192				
Gender				0.2133	0.644
Male	119	48(40.34)	71(59.66)		
Female	73	27 (36.99)	46(63.01)		
Age				0.256	1.2925
<60	60	27(45.00)	33(55.00)		
≥60	132	48(36.36)	84(63.64)		
Location				4.3688	0.037*
Colon	146	51(34.93)	95(65.07)		
Rectum	46	24(52.17)	22(47.83)		
Histological type				1.4509	0.228
Tubular	170	69(40.59)	101(59.41)		
Other^a^	22	6(27.27)	16(72.73)		
Differentiation				4.5562	0.033*
Well+Middle	153	58(49.66)	95(50.34)		
Poor	17	11(18.75)	6(81.25)		
Other^b^	22	6	16		
TNM stage				13.1541	0.004*
0+ I	*43*	11(25.58)	*32(74.42)*		
II	76	26(34.21)	50(65.79)		
III	*63*	*30(47.62)*	*33(52.38)*		
IV	*10*	*8(80.00)*	*2(20.00)*		
T				0.5275	0.468
Tis+T1+T2	49	17(34.69)	32(65.31)		
T3+4	143	58(40.56)	85(59.44)		
N				9.6676	0.022
N0	121	39(32.23)	82(67.77)		
N1a	34	14(41.18)	20(58.82)		
N1b	19	10(52.63)	9(47.37)		
N2a, b	18	12(66.67)	6(33.33)		
M				7.4030	0.007*
M0	182	67(36.81)	115(63.19)		
M1	10	8(80.00)	2(20.00)		
Preoperative CEA, ng/m1				3.0637	0.080
≦5	116	41(35.34)	75(64.66)		
>5	26	14(53.85)	12(46.15)		
Unknown	50	20	30		

* *P*<0.05;

a others: papillary adenocarcinoma, 3 cases; adeno-squamous carcinoma, 1 case; mixed adenocarcinoma,11 cases; mucinous carcinoma, 5 cases; signet ring cell carcinoma,1 case; squamous cell carcinoma, 1 case;

b others: besides tubular.

### Prognostic value of IDH1-R132H protein expression in gastrointestinal cancer

**Figure 4:**
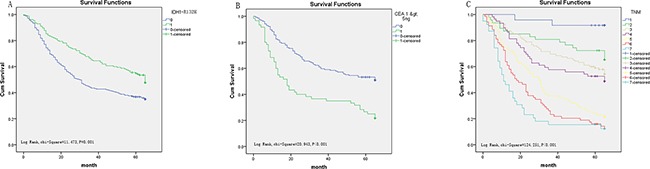
Survival curves of gastric cancer by the Kaplan–Meier method and log-rank test **A.** OS curves of high (green line, 1) and low (blue line, 0) IDH1-R132H expression. **B.** OS curves by preoperative CEA, high (green line, 1) and low (blue line, 0). **C.** OS curves by stage, TNM 0+ Ia(blue line, 1), TNM Ib(green line, 2), TNM IIa (light yellow line, 3), TNM IIb(purple line, 4); TNM IIIa (yellow line, 5); TNM IIIb (red line, 6); TNM IIIc and IV(light blue line, 7).

In the present study, we used univariate and multivariate analysis to investigate the prognostic value of IDH1-R132H expression in gastrointestinal cancer. In GC, univariate analysis showed that low IDH1-R132H expression (HR, 0.634, *P*=0.001) was correlated with poor OS, along with prognostic factors that were reported previously, including differentiation (HR, 1.639, *P*<0.001), tumor stage (HR, 1.529, *P*<0.001), tumor size (HR, 1.765, *P*<0.001), lymph node metastasis (HR, 1.642, *P*<0.001), distant metastasis (HR, 3.188, *P*<0.001), preoperative CEA levels (HR, 2.279, *P*<0.001), and CA19-9 levels (HR, 2.422, *P*<0.001). Multivariate analysis on IDH1-R132H expression, differentiation, tumor stage, preoperative CA19-9 level, and CEA level revealed that low IDH1-R132H expression (HR, 0.587, *P*=0.019), tumor stage (HR, 1.533, *P*<0.001), and preoperative CEA (HR, 2.432, *P*<0.001) correlated with poor OS (Table 4). In addition, Kaplan–Meier analysis showed that patients with lower IDH1-R132H expression, higher preoperative CEA level, and more advanced tumor stage have shorter survival time. By the log rank test, low IDH1-R132H expression (*P*=0.001), preoperative CEA level (*P*<0.001), and tumor stage (*P*<0.001) all correlated negatively with OS (Figure 4).

**Table 4:** Univariate and multivariate analysis of prognostic factors for overall survival in gastric cancer patients

	Univariate analysis				Multivariate analysis			
	HR	P >|z|	95% CI		HR	P >|z|	95% CI	
IDH1-R132H expression								
High vs Low	0.634	0.001*	0.485	0.829	0.587	0.019*	0.377	0.915
Age (years)								
≤60 vs >60	1.134	0.352	0.870	1.480				
Gender								
Male vs Female	0.892	0.425	0.674	1.181				
Histological type								
Tubular vs Mixed (Tubular and mucinous) vs Mucinous vs Signetring cell carcinoma vs others^a^	1.019	0.779	0.892	1.165				
Differentiation								
Well vs Middle vs Poor	1.639	<0.001*	1.250	2.149	1.300	0.220	0.855	1.978
TNM stage								
0+Ia vs Ib vs IIa vs IIb vs IIIa vs IIIb vs IIIc and IV	1.529	<0.001*	1.410	1.658	1.533	<0.001*	1.341	1.753
T								
Tis+T1 vs T2 vs T3 vs T4	1.765	<0.001*	1.462	2.130				
N								
N0 vs N2 vs N3	1.642	<0.001*	1.472	1.831				
M								
M0 vs M1	3.188	<0.001*	2.066	4.919				
Preoperative CEA,(ng/ml)								
≤ 5 vs ≥ 5	2.279	<0.001*	1.580	3.286	2.432	<0.001*	1.534	3.855
Preoperative CA199, (U/ml)								
≤ 37 vs>37	2.422	<0.001*	1.573	3.730	1.503	0.142	0.873	2.589

* *P*<0.05;

a others: papillary adenocarcinoma, 3 cases; adeno-squamous carcinoma, 3 cases; squamous cell carcinoma, 2 cases; neuroendocrine carcinoma,1 case; carcinoid,1 case;

b others: besides tubular.

**Figure 5:**
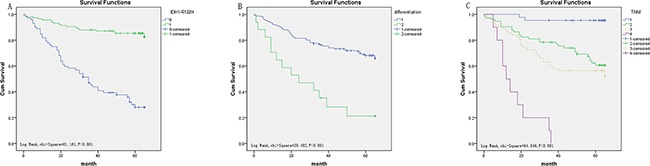
Survival curves of CRC by the Kaplan–Meier method and log-rank test **A.** OS curves of high IDH1-R132H expression (green line, 1) and low IDH1-R132H expression (blue line, 0). **B.** OS curves by differentiation, poor differentiation (green line, 2), well and middle differentiation (blue line, 1). **C.** OS curves by stage, TNM 0 and I (blue line, 1), TNM IIa (green line, 2), TNM IIIb (light yellow line, 3), TNM IIIc and IV (purple line, 4).

In CRC, similarly, univariate analysis showed that low IDH1-R132H expression (HR, 0.155, *P*<0.001) was correlated with poor OS, along with prognostic factors mentioned previously including differentiation (HR, 3.794, *P*<0.001), tumor stage (HR, 2.676, *P*<0.001), tumor size (HR, 9.889, *P*<0.001), lymph node metastasis (HR, 1.613, *P*<0.001), distant metastasis (HR, 8.031, *P*<0.001), and preoperative CEA level (HR, 2.230, *P*=0.011). Multivariate analysis on IDH1-R132H expression, differentiation, tumor stage, and CEA level revealed that IDH1-R132H expression (HR, 0.156, *P*<0.001), differentiation (HR, 2.653, *P*=0.013), and tumor stage (HR, 1.551, *P*=0.022) are independent prognostic risk factors (Table 5). Furthermore, Kaplan–Meier analysis indicated that patients with lower IDH1-R132H expression, lower differentiation, and more advanced tumor stage have a poorer prognosis. By the log rank test, low IDH1-R132H expression (*P*<0.001), differentiation (*P*<0.001), and tumor stage (*P*<0.001) all correlated negatively with OS (Figure 5).

**Table 5:** Univariate and multivariate analysis of prognostic factors for overall survival in colorectal cancer patients

	Univariate analysis				Multivariate analysis			
	HR	P >|z|	95% CI		HR	P >|z|	95% CI	
IDH1-R132H expression								
High vs low and none	0.155	<0.001*	0.090	0.265	0.156	<0.001*	0.074	0.327
Age (years)								
≤60 vs >60	1.039	0.885	0.621	1.738				
Gender								
Male vs Female	1.354	0.244	0.814	2.252				
Location								
Colon vs Rectum	1.471	0.145	0.876	2.469				
Histological type								
Tubular vs Other^a^	0.743	0.487	0.321	1.717				
Differentiation								
well + middle vs poor	3.794	<0.001*	2.044	7.043	2.653	0.013*	1.233	5.707
TNM stage								
0 + I vs II vs III vs IV	2.676	<0.001*	1.937	3.698	1.551	0.022*	1.066	2.258
T								
Tis+T1+T2 vs T3+T4	9.889	<0.001*	3.106	31.487				
N								
N0 vs N1a vs N1b vs N2a + 2b	1.613	<0.001*	1.309	1.989				
M								
M0 vs M1	8.031	<0.001*	3.970	16.245				
CEA level								
≤5 vs >5	2.230	0.011*	1.198	4.150	1.597	0.169	0.820	3.111

* *P*<0.05;

a others: papillary adenocarcinoma, 3 cases; adeno-squamous carcinoma, 1 case; mixed adenocarcinoma,11 cases; mucinous carcinoma, 5 cases; signet ring cell carcinoma,1 case; squamous cell carcinoma, 1 case;

b others: besides tubular.

## DISCUSSION

IDH1 is a NADP-dependent enzyme that catalyzes the decarboxylation of isocitrate into alpha-ketoglutarate and provides the needed cytosolic NADPH [21, 26]. IDH1 functions as a tumor suppressor since its inactivation contributes to tumorigenesis [22]. IDH1 mutations are common in gliomas and have a positive impact on prognosis [27, 28]. In contrast, IDH1 mutations predict poor prognosis in myeloproliferative neoplasms and myelodysplastic syndrome [29]. Although IDH1 mutations have also been found in GC [30], the association between IDH1-R132H expression and GC outcome has not been examined. In this study, we examined IDH1-R132H expression in gastrointestinal cancers by immunohistochemistry in human tissue. We found that the expression of IDH1-R132H correlated with GC, indicated by tumor stage, especially lymph node metastasis. In CRC, the expression of IDH1-R132H was correlated with location, differentiation, tumor stage, lymph node metastasis and distant metastasis. Then, we also examined IDH1-R132H expression in various gastrointestinal cancer cell lines using western blot analysis. Our findings indicated that the expression of IDH1-R132H in well-differentiated cell lines is higher than that in poorly differentiated cell lines, which is consistent with our immunohistochemistry results in human tissue. In both GC and CRC, low expression of IDH1-R132H was correlated with poor OS. A recent study reported that IDH1-R132H expression correlates positively with angiogenesis and cell proliferation in glioma samples [31]. The mechanism underlying the impacts of IDH1-R132H expression on survival in various malignancies are not clear, and further studies are required to investigate these differences.

In the present study, IHC in TMA revealed that IDH1-R132H expression was lower in cancerous tissues than in normal and benign tissues. Furthermore, we found that IDH1-R132H expression correlated negatively with tumor stage in both GC and CRC. In addition, IDH1-R132H expression correlated negatively with OS in GC patients according to both univariate and multivariate analysis.

IDH1 encodes two tricarboxylic acid cycle (TCA) enzymes, fumarate hydratase and succinate dehydrogenase, which help maintain steady-state levels of the TCA metabolites malate and fumarate [32, 33]. The R132H substitution represents around 90% of IDH1 mutations [34, 35], which allows IDH to promote the transformation of α-ketoglutarate to 2-hydroxyglutarate, ultimately leading to tumorigenesis. The product of the IDH1 forward reaction, α-ketoglutarate, is an intermediate in the TCA [36, 37]. A recent study observed that TCA cycle impairment might support tumorigenesis by interfering with the hypoxia-inducible factor 1α pathway [38, 39]. Furthermore, IDH1 catalyzes the production of NADPH, the levels of which limit the growth and survival of cancer cells. IDH1 mutations decrease the affinity of the IDH1 active site for isocitrate while increasing it for NADPH [37]. NADPH provides redox power to neutralize oxidative stress, which is critical in situations of metabolic stress for cancer cell survival. In addition, as a co-enzyme for anabolic enzymes, NADPH plays an important role in the generation of new building blocks to maintain cell growth and proliferation [21, 40]. Therefore, IDH1 mutations might be involved in various regulatory pathways of gastrointestinal cancer, making IDH1-R132H a prospective therapeutic target. Indeed, some reports have suggested that small-molecule inhibitors targeting the IDH1-R132H mutant protein represent a viable treatment strategy [37]. Popovici-Muller et al. reported compound 35 as a potent inhibitor of IDH1-R132H [41]. A recent high-throughput screening identified compound AGI-5198 as another potent small-molecule inhibitor of IDH1-R132H [42].

In this research, we investigated the association between the expression of IDH1-R132H protein and clinical parameters in gastrointestinal cancers. One limitation of our study is that we did not evaluate IDH1 mutation by direct gene sequencing to confirm our results. Moreover, we only used IHC and western blot to determine the expression of IDH1-R132H at the protein level. Although more analyses, such as studying IDH1-R132H at the mRNA level, could help to better understand the mechanisms underlying the role of IDH1-R132H in gastrointestinal cancer, our results show that low IDH1-R132H expression may be used as an independent prognostic marker in gastrointestinal cancers.

## MATERIALS AND METHODS

### Human tissue samples and patient clinical information

We obtained 925 tissue specimens from 755 patients, including 526 stomach tissues (412 cancer, 42 matched normal surgical margins, 13 low-grade intraepithelial neoplasia, 34 high-grade intraepithelial neoplasia, 13 chronic gastritis, and 12 intestinal metaplasia), and 399 colorectal tissues (192 cancer, 128 matched normal surgical margins, 43 low-grade intraepithelial neoplasia, and 19 high-grade intraepithelial neoplasia, and 17 chronic colitis). The samples were formalin-fixed and paraffin-embedded (FFPE). A total of TMAs were collected at the Affiliated Hospital of Nantong University from 2002 to 2009. Patient information was obtained from medical records and included age, gender, differentiation grade, tumor stage, histological type, CA19-9 levels, preoperative serum CEA. The guideline of the 7th edition of TNM staging in malignant tumors was used to determine tumor stage. Before surgery, all of the patients had not received chemotherapy, radiotherapy, or immunotherapy. We defined the period from the initial diagnosis until death as the 5-year OS. The last follow-up date on which the patients were alive was censored from the analysis. All patients provided written informed consent. The study protocol was approved by the Ethics Committee of the local hospital, and was conducted according to authorized guidelines of the Affiliated Hospital of Nantong University.

### TMA construction and IHC analysis

As previously described [43], a Tissue Microarray System was presented to generate TMA for further IHC analysis in the Department of Pathology, Affiliated Hospital of Nantong University. In brief, TMA slides were incubated using a mouse, monoclonal, anti-human IDH1 R132H antibody (5 μg/mL, 10389; IBL, Japan). The Envision+TM peroxidase kit (Dako, Carpinteria, CA, USA) was used as the secondary antibody.

All staining results were observed and scored blindly; at the same time, independent evaluations were performed. The expression of IDH1-R132Hwas scored according to the staining intensity as follows: 0 (−, none), 1 (+, mild staining), 2 (+ +, medium staining), or 3 (+ + +, intensive staining). The product of intensity scores and percentages was calculated ranging from 0 to 300 and defined as the final IHC score.

### Cell lines and cell culture

Four human GC cell lines HGC-27, MKN-28, MKN45, MGC80-3 and four human CRC cell lines were obtained from the Chinese Academy of Sciences (Shanghai, China). All lines were maintained in RMPI-1640 (Thermo, NY, USA) and supplemented with 10% fetal bovine serum (FBS, Gibco, CA, USA). All cell lines were cultured in 5% CO^2^ at 37°C.

### Western blot analysis

As previously described [44], western blot was carried out as follows: the cell lines were respectively digested in 0.25% Trypsin-EDTA (Gibco, NY, USA) and lysed in lysis buffer (Beyotime Institute of Biotechnology, Nantong, China) for 15 min on ice and centrifuged at 13,000g for 15 min at 4°C. Total concentrations were determined using the BCA method. The total protein samples were subsequently run through SDS-polyacrylamide gels (10%) and transferred onto PVDF. Binding was blocked using 5% bovine serum albumin (BSA) at room temperature for 2h and immunoreactivity was performed using the following primary antibodies: mouse anti-human IDH1-R132H antibody (1:500 dilution, 10389; IBL, Japan), and rabbit anti-GAPDH (1:2000 dilution, Goodhere, Hangzhou, China). The membranes were incubated with goat anti-mouse IgG antibody or goat anti-rabbit IgG antibody labeled with HRP-conjugated goat anti-rabbit secondary antibody (1:2000 dilution; Abcam, UK). After further washing, the membranes were scanned using an enhanced chemiluminescence system (ECL, Beyotime Institute of Biotechnology) and the data were analyzed by densitometry.

### Statistical analysis

The cutoff point for statistical analysis [43, 45] was determined in terms of OS using the X-tile software program. The relationships between clinical parameters and the expression of IDH1-R132H were calculated using χ^2^ tests. Kaplan-Meier analysis was used to evaluate survival curves and log-rank test was conducted to verify them. Additionally, we used univariate analysis and multivariate analysis to evaluate the prognostic value for patients with GC and CRC. STATA 12.0 (Stata Corporation, College Station, TX, USA) and SPSS 20.0 software (IBM Corporation, Armonk, NY, USA) were used for data analysis. For all tests, *P*<0.05 was considered to be statistically significant.
